# Family-portraits for daphnids: scanning living individuals and populations to measure body length

**DOI:** 10.1007/s10646-015-1490-0

**Published:** 2015-06-06

**Authors:** Annika Agatz, Monika Hammers-Wirtz, Andre Gergs, Tanja Mayer, Thomas G. Preuss

**Affiliations:** University of York, Heslington, York, UK; Research Institute for Ecosystem Analysis and Assessment (gaiac), Aachen, Germany; Institute for Environmental Research at the RWTH Aachen, Aachen, Germany; Dr. Knoell Consult GmbH, 51377 Leverkusen, Germany; Bayer CropScience AG, Monheim am Rhein, Germany

**Keywords:** Method development, Structure analysis, Size, Non-destructive measurement, Aquatic invertebrates

## Abstract

A method has been developed and tested to determine the body length of living daphnids. The purpose of the method was the simple, accurate, repeatable, quick, and to the living organism, harmless measurement of body length of all individuals in a population to enhance the capability of observing population development over time. Generally, organisms are transferred to a petri dish and temporarily fixed by removing access medium. A picture of the petri dish is taken using an ordinary flatbed scanner. Pictures are manually analysed with purposely developed software. We found no significant impact of the method on either individual performance (growth and reproduction) or population development (abundance and structure) of daphnids in comparison to the previously used method for data gathering (sieving, counting and length measurement of a subsample via microscopy). The disadvantage of our method, an increased demand in time for picture analysis, is negligible compared to the advantages this method has. Data generated with the new method do represent the population structure more accurately than those data generated with the previously used method. Scanning organisms does also allow a retrospective quality control for generated data as pictures can securely be stored. The quality of the pictures is furthermore sufficient to include additional endpoints to the analysis (e.g., number and size of aborts, number and size of eggs in the brood pouch, spine length). Here, we present, test and discuss an alternative approach to automated image analysis for data gathering in single and multiple individual and species experiments.

**Electronic supplementary material:**

The online version of this article (doi:10.1007/s10646-015-1490-0) contains supplementary material, which is available to authorized users.

## Introduction

It has frequently been suggested that the standard reproduction test with daphnids might fail to generate sufficient data to predict effects occurring at the population level due to shortcomings in generated data (Martin et al. 2014). Hammers-Wirtz and Ratte (2000) for example demonstrated that the outcome of standard reproduction test for *Daphnia magna* (OECD 1998) might in some cases not be sufficient to predict population level effects because impacts on the fitness of the offspring (reflected in their body size at birth) are not assessed. They proposed to either expand the experimental duration to assess impacts to the next generation or at least measure the body size of the offspring (Hammers-Wirtz and Ratte 2000). In recent years, the effect assessment on subsequent generations (Brennan et al. 2006, Massarin et al. 2010, Volker et al. 2013, Jacobasch et al. 2014) and the measurement of growth (Pieters and Liess 2006, Agatz et al. 2013, Jacobasch et al. 2014) and offspring size (Ebert 1993, Boersma 1997, Guinnee et al. 2007) found its way into the research driven effect assessment. The measurement of size can usually be conducted via the destructive mass assessment (determining dry weight) at the end of an experiment or via direct and indirect length measurement. Direct length measurement would be the determination of length under the microscope, whilst indirect measures are made by sieving through a sieve cascade with different mesh sizes (Hammers-Wirtz and Ratte 2003) or image analysis tools via photos taken from the test vessel (Pieters and Liess 2006). Destructive mass assessment does not allow for multiple measurements on individuals. Non-destructive methods have, to our knowledge, not been tested regarding impacts on individual performance and can be time demanding. Indirect measures have only been used for population experiments whereas microscopy is usually conducted for tests at the individual level. A subsample of all replicates is usually used when measuring the body length of individuals under the microscope (Hammers-Wirtz 2002). This practical decision however introduces uncertainties into the experimental analysis. For *Daphnia* it is known that offspring size is determined by food availability for mothers and their body size (Gliwicz and Guisande 1992; Enserink et al. 1995; Boersma 1997, Cleuvers et al. 1997; Gabsi et al. 2014). Measuring only a subsample and referring to the average of the measurement introduces analytical uncertainty. Furthermore, accurate statistical analysis can be difficult with data gathered from different subsamples over time due to a very variable representativeness of the gathered data for the whole experiment. If for example the body length of ten offspring produced by all replicates within 1 day is measured, this subsample can be generated by only one or all replicates.

Conducting population experiments can overcome the challenges a reproduction test raises regarding the extrapolation of effects to the population level. Even when conducting tests at the population level effects can remain undetected if population structure is not investigated (Gergs 2013). Measuring the length of all individuals in a population to gather information on population structure becomes even more challenging than for tests at the individual level. Populations consist of individuals of all sizes and old and/or weak individuals die whereas new individuals are produced constantly. Hammers-Wirtz and Ratte 2003 proposed the classification of individuals in a population into three size classes to allow the analysis of population structure. Size classes were defined as organisms within one of three sieves after sieving the whole population through a sieve cascade of increasing mesh size; separating neonates, juveniles and adults. Alternatively, an automated system for the classification of populations into four size classes was used (Liess et al. 2006). This automated system is more advanced and uses a sort of size measurement, by estimating the size of a daphnids after combining three consecutive pictures, removing non-moving objects, removing noise and adding data gaps (Liess et al. 2006). Both methods however do not allow measuring the actual length of individuals and the minimal and maximal length of organisms in the population; all valuable information for effect assessment. A non-destructive method to measure the length of all individuals in a reproduction test or in population experiments would not only benefit the interpretation of the frequently undertaken destructive mass assessment at the end of an experiment but would allow detailed assessment of individual growth and population structure. Scanning living individuals with subsequent picture analysis was identified as potential method to measure *Daphnia* length. Reproduction tests (Agatz et al. 2013) and whole population experiments (Agatz et al. 2012; Agatz and Brown 2013; Gergs et al. 2014; Simon et al. 2015) using this approach have been conducted. The method has also been used to refine size classes of daphnids (Gergs et al. 2014) and to measure the length of daphnids as prey in predation experiments (Gergs and Ratte 2009; Gergs et al. 2010). Nonetheless, the method has not been thoroughly reported for potential users and the evidence for the non-destructive nature of this method was unpublished.

Here, we present details on the scanning method. We compare data generated with this method to data generated using another method, discuss advantages and disadvantages, and illustrate the main influencing factors for the outcome of length measurement analysis with the scanning method.

## Method

### Generating pictures

Organisms and some of their medium are transferred from their beaker onto a petri dish using a sieve (mesh diameter 0.27 mm). Organisms are fixed to the petri dish by carefully removing excess water until no movement of the organisms is observed, while leaving enough water around the individuals to continue filtering water. The petri dish is placed at the scanner and a picture is taken using the preliminary set scanner settings. So far the method of body length measurement has been calibrated using the colour image scanners CanoScan 8800F and 9000F from Canon at 1200 dpi and the CanoScan D-1250U at 300 dpi.

A video demonstrating the whole process of generating a picture using a group of different sized daphnids is provided in the Online Resource 2.

### Calibrating the software to the scanner settings

For the accurate measurement of body length it is necessary to calibrate the software to the scanner and the scanner settings chosen to generate the picture. To do so, millimetre scale has been placed face down on the bottom of a petri dish and was scanned using the identical scanner setting as chosen for scanning the daphnids. The software incorporates a measurement procedure for standards (pictures of millimetre scale) which can be used to identify the number of dots per inch (dpi) for a chosen length (for example 1 mm). This standard measure can be saved to allow measurements of all pictures taken with the same scanner and scanning settings without further need of length calibration.

### Picture analysis

Purposely developed software is used to manually measure the body length of individuals in the picture. The software has been developed using Delphi (Embacadero RAD Studio XE2). A version of the software is provided in the Online Resource 3.

Overall, the software allows opening the picture in its original size, the user can identify the standard to measure against and needs to manually identify the starting and ending point of a measurement by clicking on it. The processed picture (indicating the measurement) can be saved as jpg-file. Generated data can be stored as Excel readable csv-files. A demonstration of the software is provided in the Online Resource 4.

## Method testing

### Finding the right resolution for the scanning process

The body length of three daphnids of three different size classes (one neonate, one juvenile and one adult) was measured using a microscope (WILD Heerbrugg). Subsequently, the individuals were transferred to a petri dish and pictures were taken as described above at four different settings for the resolution of the scanner (360, 720, 1200 and 2400 dpi). Pictures were analysed using the purposely developed software (Online Resource 3) and measured body length was compared to the measurements generated using the microscope (Mayer 2008).

### Development of individual daphnids over time

The body length of 20 neonates (<24 h) was measured 2–3 times a week for a total of 30 days. Body length was determined using the microscope for half of the organisms. The remaining organisms were scanned and body length was measured via picture analysis. Organisms were treated identically except for the method used to generate length data. Individuals were kept individually in 90 ml M4-medium (Elendt 1990) at 20 ± 1 °C at a 16 h:8 h light–dark-rhythm and were fed daily with 0.1 mg total organic carbon of the green algae *Desmodesmus subspicatus*.

### Population development over time

A population experiment was conducted over 5 weeks to determine whether frequent data acquisition itself and the type of data acquisition via scanning and microscopy influence the population development. The experiment consisted of four treatments with three replicates each. Three treatments (scanning (1), sieving with microscopy (2) and sieving without microscopy (3)) were set up to be semi-static; thus populations were handled frequently. Three times a week populations were transferred to fresh medium via sieving. During this process data acquisition took place every time (treatments 1 and 2) or only twice in total (day 19 and 35, treatment 3). Next to these semi-static populations treated with frequent sieving the experiment consisted of populations where medium had not been changed (treatment 4) and populations were not handled frequently. Data acquisition for treatment 4 took only place twice (day 19 and 35) via sieving with microscopy.

All populations were started with five neonate (<24 h) and three adult (2.9–4.0 mm in length) daphnids, kept in 950 ml M4-medium (Elendt 1990) at 20 ± 1 °C at a 16 h:8 h light–dark-rhythm, as proposed previously (Hammers-Wirtz 2002). Populations were fed daily with 0.5 mg total organic carbon of the green algae *Desmodesmus subspicatus* (per day and population).

Four sieves differing in their mesh diameter were used to separate individuals in four categories. These categories were proposed to be adults (age > 6 days, body length > 2.6 mm), juveniles (age > 2 days, body length 1.4–2.6 mm), neonates (age < 2 days, body length < 1.4 mm) and aborts. Sieves used were scanned and measured. Mesh diameters were found to be 1.36, 0.66, 0.40 and 0.27 mm for the sieves for adults, juveniles, neonates and aborts, respectively. A cascade of all sieves was used on days of data acquisition for treatments using microscopy for length measurement to allow the immediate count of individuals belonging to the different size classes prior to length measurement of a subsample (n ~ 10). Only the sieve with the smallest mesh size (Ø 0.27 mm) was used to collect the whole population for scanning and for transfer to fresh medium without data acquisition (Mayer 2008).

### Identifying the actual size classes separated by using the sieve cascade

After the observation of population development was completed individuals of one population were classed using the sieving with the sieve cascade and the scanning method. All groups and their corresponding sieves were scanned to compare the presumed and actual separation via the sieving method (Mayer 2008).

### Identifying some causes of inaccuracy using the scanning method

A series of more than 200 pictures (each consisting of one mother with their offspring <24 h) were generated for an independent reproduction test. Pictures were taken and analysed as described above in consecutive order within a period of 10 h. Analysis of all pictures was conducted by two analysts. The main difference between both analysts was the familiarity with the method (no prior experience with this analysis vs. more than 3000 pictures analysed with the method).

### Statistical analysis

Two-way analysis of variance (ANOVA) was performed with the data on individual growth and reproduction and the total population abundance. The Shapiro–Wilk test for normal distribution and the Levene-Median test for equal variance were performed prior to the ANOVA. The all pairwise multiple comparison was conducted following the Holm-Sidak method. All statistical analysis was performed using SigmaPlot 12.3.

## Results and discussion

### Finding the right resolution for the scanning process

**Fig. 1:**
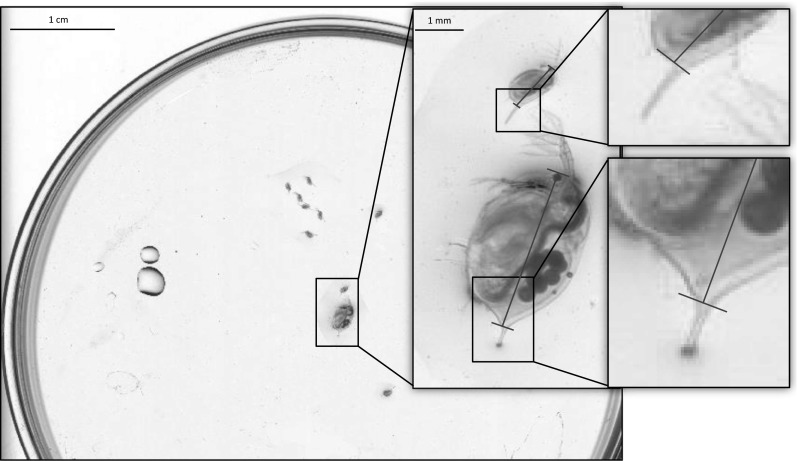
Picture of a mother and her offspring (<24 h) of *Daphnia magna* taken with a flatbed scanner at a resolution of 1200 dpi. *Red lines* illustrate the defined body length (Color figure online)

The test revealed that at 360 dpi the picture quality was not high enough to identify both the spine and the base of the spine. We therefore disregarded this resolution from further analysis as the body length of daphnids (defined as the diagonal measurement from the top of the eye to the apical base of the spine) illustrated in Fig. 1 could not be measured. At 720 dpi the resolution of the picture is not high enough to accurately determine body length. Especially whilst measuring neonates, a high deviation in body length (>9 %) was found when comparing the results to measurements made using a microscope. Increasing the resolution to 1200 dpi reduced the deviation between both measuring methods (<2.3 %). A further increase in the resolution to 2400 dpi did not result in another reduction in the deviation between both measuring methods but rather increased the deviation again (Online Resource 1, Table 1) accompanied with an increased duration of the scanning process. Increasing the resolution provoked a decrease in picture quality as increasing duration of the scanning process allowed the manifestation of minimal movement of the organisms. Our analysis suggests the use of a scanner-resolution of 1200 dpi. An example of such a picture is given in Fig. 1. However, a good balance between speed of the scanning process (reducing the chance of organisms being stressed) and picture quality (influenced by manifestation of minimal movement) is highly dependent on the hardware used. We recommend using the highest resolution possible without manifestation of movement of individuals; whereas it is important not to manipulate movement of individuals by increased removal of medium for fixation on the petri dish.

Since minimal movement of individuals (and with this movement of filtering appendices inside the carapax) is recorded during the scanning process generated pictures have the advantage to disclose additional information. The movement of filtering appendices (shown in the picture as rainbow-coloured areas) combined with the colour of the algae in the upper part of the gut can be used to distinguish living and dead individuals. A detailed illustration is shown in the Online Resource 1, Fig. 1. The picture quality also allows the investigation of further endpoints. It is for example possible to count and measure offspring in the brood pouch or measure spine length and other morphological characteristics.

### Development of individual daphnids over time

There was no statistically significant difference in body length and reproduction when comparing the microscopy and scanning method (Online Resource 1, Fig. 2) with the exception of a single significant difference in body length on day 25 (Two-way ANOVA, Holm-Sidak method, p = 0.045). This difference is however very low and is not observable at any other time point. This experiment illustrates that handling procedures involved in scanning does not alter individual behaviour in terms of growth and reproduction differently than those procedures involved in the usually used method for body length determination (placing individuals under the microscope and fixating them by removing excess water).

### Population development over time

**Fig. 2:**
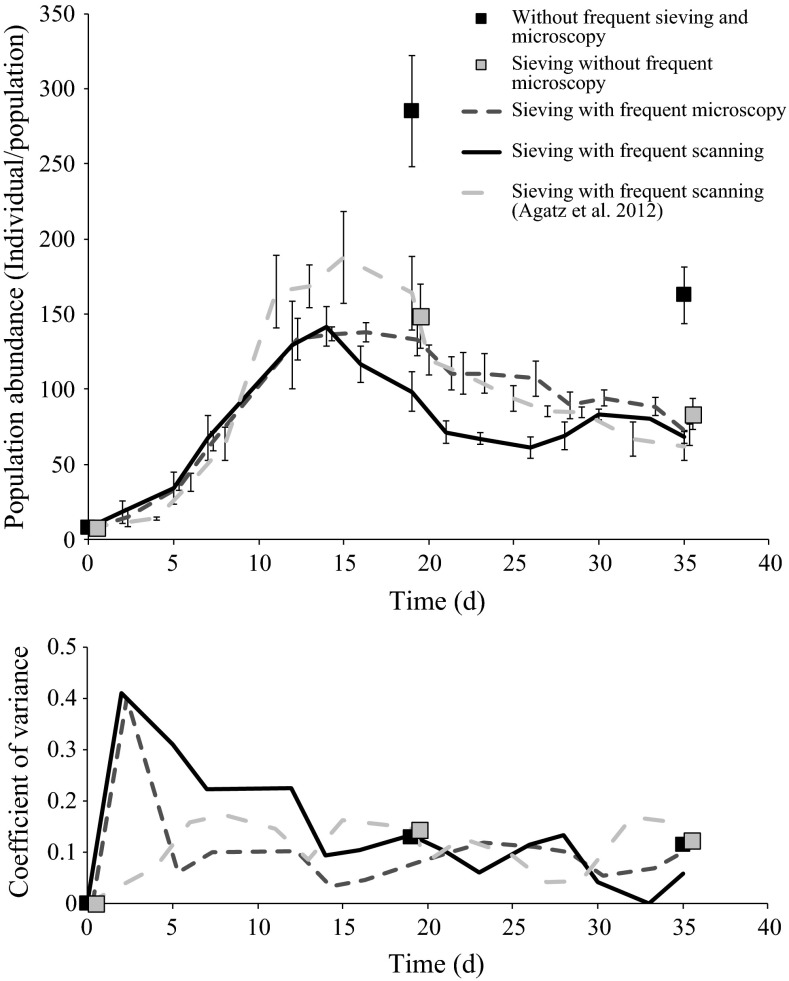
Average total abundance of *Daphnia magna* populations (*top*) and the corresponding coefficients of variances (*bottom*) over time. Shown are the four treatments of the Population test and the control treatment of another population test (Agatz et al. 2012)

Sieving itself seems to have an impact on population development. Populations only disturbed twice within the experimental duration by sieving through a sieve cascade showed on both days of data acquisition (days 19 and 35) much higher total population abundance as all other populations (Two-way ANOVA, Holm-Sidak method, p < 0.001 for all comparisons). If sieving through the smallest sieve took place three times a week to transfer the population to fresh medium total abundances were significantly smaller on both days of data acquisition (Fig. 2, top). Whether this difference in total abundance is down to destruction of the population through sieving *per se* cannot be ruled out. However, it is more likely that populations not sieved frequently reached higher abundances because they were kept in a static system without medium change. Not renewing the medium likely increases the food availability for the population through both a lack of removing not eaten algae and growth of bacteria. It has frequently been observed that daphnids can graze on bacteria (Degans et al. 2002; G-Toth et al. 2002).

Whether one sieve or a sieve cascade is used to transfer populations to fresh medium does not show any impact (Two-way ANOVA, Holm-Sidak method, p > 0.46) on the total population abundance (Fig. 2, top; sieving without frequent microscopy vs. sieving with frequent microscopy). Thus, frequent data acquisition *per se* does not disturb population development in semi-static experiments; making high temporal-resolution observations possible without reducing the potential of lab experiments reflecting field populations.

Comparing data acquisition via scanning and microscopy in population experiments (Fig. 2, top; sieving with frequent microscopy versus sieving with frequent scanning) indicates that scanning might induce a faster decline of the population abundance to the population capacity at equilibrium. The abundance of populations being sieved is significantly lower than the abundances for those being measured by microscopy from day 16 to day 28 (Two-way ANOVA, Holm-Sidak method, p < 0.016). Population abundance at equilibrium however does not show any differences occurring from the type of data acquisition used (Two-way ANOVA, Holm-Sidak method, p > 0.101).

Neither treatment had an impact on the coefficient of variance within the experiment (Fig. 2, bottom), clearly indicating that the choice of data acquisition does not induce the need to adjust the number of replicates needed.

Figure 2 includes the control treatment of an independently conducted population experiment (Agatz et al. 2012) which used exactly the same experimental conditions and the same method of data acquisition as the treatment “sieving with frequent scanning” as in the experiment conducted by Mayer (2008). These data were not statistically tested over all time points as the actual time points of investigation mostly differed to all other treatments. For the 2 days of interest (day 19 and 35) an interpolation of the two surrounding days of data acquisition (18 and 20; 34 and 36) was used for comparison.

The added data set shows significantly higher maximum population abundance and higher abundances throughout the population decline until reaching the same population capacity at equilibrium as all other populations. Differences are likely driven by maternal status of individuals when populations were started (e.g., reproductive stage, body length, status of energetic reserve). The relative decline towards the equilibrium however is similar to that of the other populations treated to scanning. This data set has been included to illustrate that fluctuations between experiments can occur throughout the population development towards the equilibrium. More importantly it shows that an impact of scanning on population abundance might not be ruled out but does, if at all having an impact, influences population development consistently. Meaning that treatments are comparable to their control and experiments are comparable to each other.

**Fig. 3:**
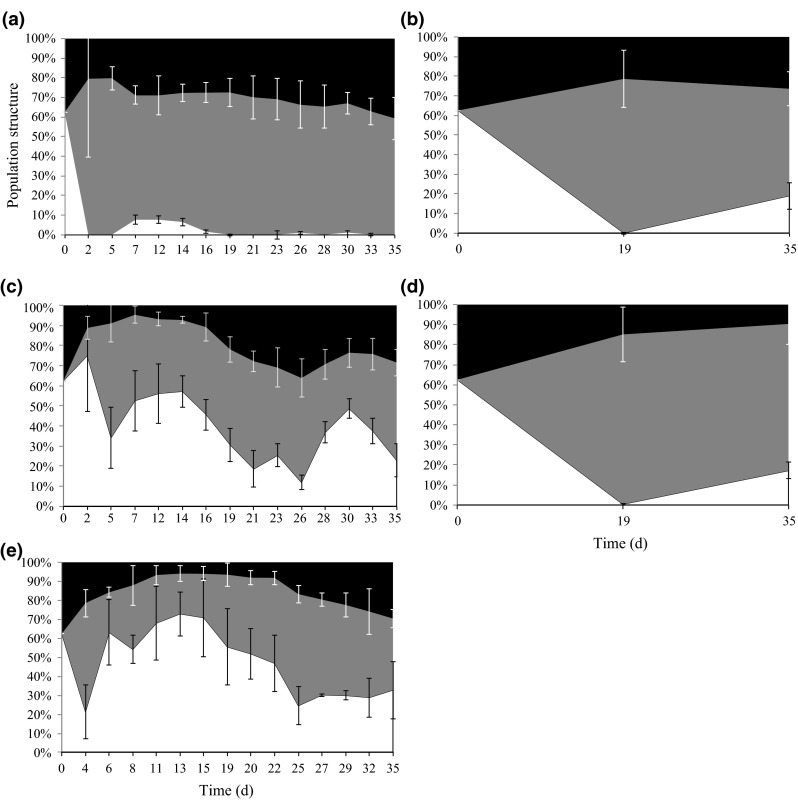
Population structure (% neonates (*white*), juveniles (*grey*) and adults (*black*)) of the total population abundance of *Daphnia magna* over time. Shown is the average (±SD) of the four treatments from the population test **a** sieving with frequent microscopy, **b** sieving without frequent microscopy, **c** sieving with frequent scanning, **d** without frequent sieving, **e** sieving with frequent scanning of another population test (Agatz et al. 2012). Adults (body length > 2.6 mm); juveniles (body length 1.4–2.6 mm); neonates (body length < 1.4 mm)

Sieving the population through a sieve cascade and counting individuals in each sieve certainly requires some time but allows the investigation of the population structure by classifying certain groups of the population. The comparison of population structure shows that frequent change of medium does not influence the composition of a population (Fig. 3b vs. d). The type of data acquisition on the other hand seems to have a strong impact on the population structure (Fig. 3a vs. c, e). This clear difference however is an artefact of the different methods of categorising individuals into size classes and is not an indication of a shift in population structure caused by the type of data acquisition (an explanation is given in the next paragraph).

### Identifying the actual size classes separated by using the sieve cascade

**Fig. 4:**
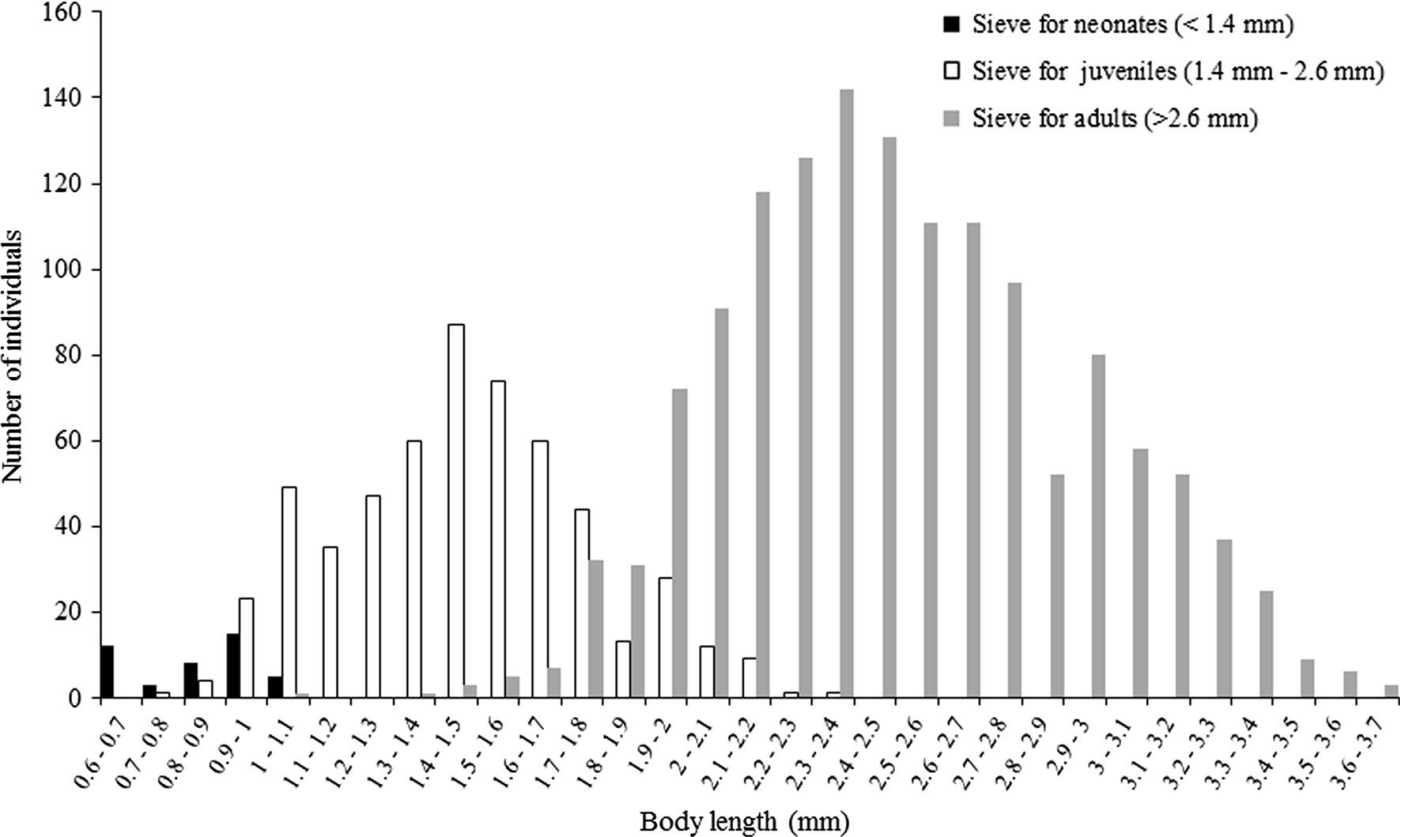
Body size distribution of a *Daphnia magna* population after 42 days measured with the scanning method after separation according to the sieving method

Figure 4 shows that individuals caught in the sieves for neonates, juveniles and adults do not necessarily have the body length required to be categorised as such individuals. Overall analysis of all individuals in the three sieves with the scanning method (Fig. 4) revealed that 100 % of individuals ending up in the sieve for neonates are in fact neonates. Looking at the sieves for bigger individuals, however, only 39.9 and 37.9 % of juveniles and adults, respectively, ended up in the correct sieves. The remaining 60.1 % of organisms in the sieve for juveniles are in fact neonates according to their measured body length and the definition of neonates measuring less than 1.4 mm. Even 2 individuals found in the sieve for adults were neonates according to their length. The other 62.1 % were in fact juveniles measuring between 1.4 and 2.6 mm. One could argue that a simple solution would be to re-categorise the size classes caught in the sieves according to actually measured body length taking into account the mesh diameter. However, there are constrains to the re-categorisation of groups. Size classes were mainly meant to represent a classification of a population into “functional groups” (neonates, juveniles and adults) to allow a sensible measure for population structure. Sieves with other mesh sizes need to be used to maintain the categorisation into functional groups which, to our knowledge, cannot be obtained. Nonetheless, other sieves will not prevent individuals ending up in the “wrong” sieve. Generally, random positioning of daphnids towards the sieve and the fact that daphnids are not perfectly spherical leads to overlapping size distributions in the sieves. Furthermore, true treatment effects manifested in morphological changes (e.g., development of a head capsule, alteration in spine length) could lead to individuals ending up in the wrong sieves; ultimately leading to effects being misinterpreted as a demographic shift rather than morphological plasticity.

Overlapping size distribution occurs to such an extent that a precise classification via sieving is not possible. This could result in a high variability between replicates and an increased chance of differences in population structures between treatments thoroughly reflecting errors in categorisation rather than true treatment effects. Intensification of the sieving process (e.g., multiple rinsing of the sieves with medium) could be a solution to reduce the amount of overlapping size distribution but will increase the handling time, will most certainly increase the stress for the organisms and does not allow distinguishing demographic shifts and morphological plasticity.

Using the scanning method furthermore has the advantage that it can reduce handling time. No individuals need to be counted during the actual experiment for data acquisition providing the possibility to increase the number of replicates or treatments within one experiment. Generating the data via picture analysis on the other hand can increase the overall duration of an experiment. Depending on the number of pictures and population abundances, time spend to analyse pictures might be higher than time saved not sieving through a sieve cascade and counting on the go. The advantage is that picture analysis can be conducted at any time, pictures can be stored for quality control and reanalysis, and picture analysis provides the opportunity to investigate other endpoints. The number of reproducing mothers and the number of eggs per mother can be measured and could be used to predict population growth. Such measures increase the information an experiment can deliver and increases the benefit of whole population experiments. Enabling a better understanding of population development in general and widen the potential to assess changes in populations from biotic and abiotic stresses.

### Identifying some causes of inaccuracy using the scanning method

The comparison of the results from both analysts (experienced vs. unexperienced) revealed that it is of advantage to get familiar with this method to reduce the coefficient of variance for the measurements and thus increase the statistical power for experiments. Comparing the measured averages and standard deviations for neonates in the first 20 pictures revealed that the coefficient of variance is twice the coefficient of variance for an experienced analyst. Nevertheless, this experiment showed that unexperienced analysts can reach a relatively low coefficient of variance to start with (Online Resource 1, Fig. 3). The actual coefficient of variance was 0.029 ± 0.013 and 0.058 ± 0.019 for the experienced and unexperienced analyst, respectively.

Looking at the coefficient of variance for all pictures consecutively analysed within a period of 10 h shows a further difference between the experienced and unexperienced analyst (Online Resource 1, Fig. 4). For the experienced analyst the coefficient of variance remains fairly stable over time with an overall average of 0.032 ± 0.013 and a maximum of 0.075. For the unexperienced analyst the coefficient of variance increases over time and reaches on average of 0.085 ± 0.033 with a maximum of 0.244. An inconsistency of picture analysis over time (i.e., picture number) exacerbates the analysis of the whole experiment especially when comparing different treatments over time. This analysis indicates that experience, most likely determined by the concentration level to click at the accurate spot is an important factor for consistency in accuracy.

A further factor influencing the analysis is the definition of body length itself. Usually, body length for daphnids is defined as the diagonal measurement from the top of the eye to the apical base of the spine as illustrated in Fig. 1. A deviation from this definition for example by measuring from the top of the eye to the end of the gut might not necessarily change the outcome of one experiment (because comparison is made against the own control) but complicates the comparison of the results with other experiments. Such a deviation can potentially lead to interpretations of the cause of differences which actually are not present.

A comparison of the measured body length of mothers in all pictures analysed using both mentioned definitions for body length show a constant difference of the measured length of 0.13 mm (Online Resource 1, Fig. 5).

## Conclusion

Using the scanning method gives the possibility to increase the number of treatments within one experiment as handling time of populations is decreased. Users are able to accurately determine the population structure accounting for all individuals in the population. They are no longer restricted to group the individuals in a population into age/size classes specified by the sieves used, and they have the opportunity to greatly enhance the amount of information gathered in their test. Users have the chance to fit the type of endpoint to be measured to the underlying research question without having to change the method to be used or the need of changing the analysis programme.

There are disadvantages compared to other methods. Predominantly the additional time needed for picture analysis and a chance of analytical differences between two experimentalists could be seen negative. Automated image analysis as proposed frequently (Færøvig et al. 2002; Hooper et al. 2006; Liess et al. 2006) avoids these factors, but introduces other potential disadvantages. The relative high demand of special equipment (light sources, cameras, high performance computer) compared to an ordinary flatbed scanner, the demand of knowledge on underlying algorithm and the capability of adjusting these in order to alter the system towards specific research questions, and the uncertainty in data obtained caused by the system algorithms are examples.

We can and will not advise what method is best to be used as this is thoroughly determined by the underlying research question to be answered and/or the frequency an experiment is to be carried out, but rather present an alternative approach. The presented method is suitable to be used in both single organism experiments and whole population experiments and can easily be adapted for other species of interest and communities.

## Electronic supplementary material

Online Resource 1 (PDF 339 kb)

Online Resource 2 (MP4 88495 kb)

Online Resource 3 (ZIP 672 kb)

Online Resource 4 (PDF 1108 kb)
