# Supplementary material for: Family-portraits for daphnids: scanning living individuals and populations to measure body length
Source: Ecotoxicology. 2015 Jun 6;24(6):1385–94. doi: 10.1007/s10646-015-1490-0 (PMC4515252; doi:10.1007/s10646-015-1490-0)
Supplement: Supplementary file 1 — Online Resource 1 (PDF 339 kb) [file 10646_2015_1490_MOESM1_ESM.pdf]

Family-portraits for *Daphnids* – Scanning living individuals and populations to measure body length

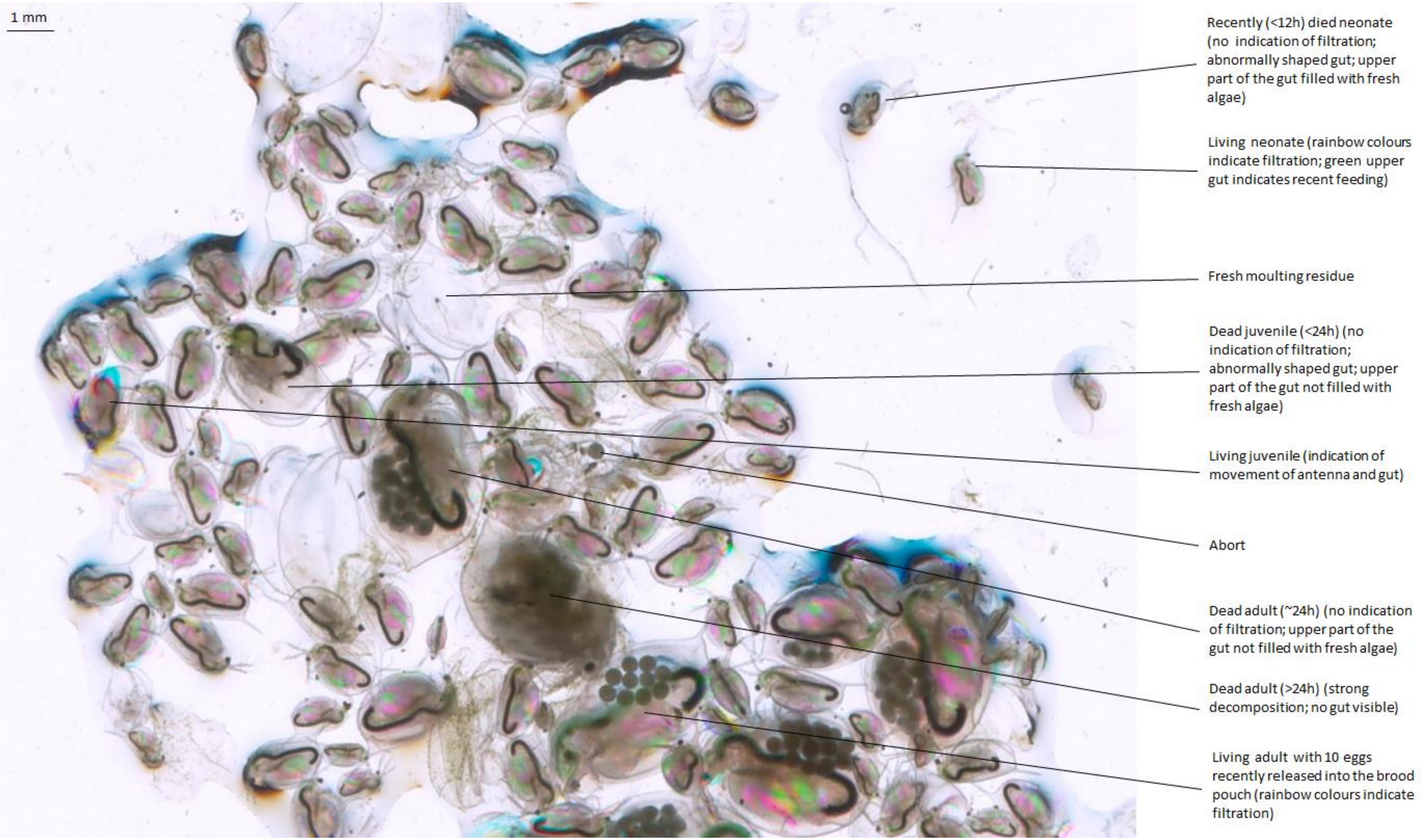

Figure 1: Illustration and explanation of a scanned population of *Daphnia magna* at a resolution of 1200 dots per inch.

Table 1: Comparison of body size measurements (mm) of Daphnids using microscopy and picture analysis.

	Microscopy	Picture analysis		
		720 dpi	1200 dpi	2400 dpi
Adult	2.80	2.75 (1.79)[a]	2.79 (0.36)[a]	2.77 (1.07)[a]
Juvenile	2.16	2.13 (1.39)[a]	2.11 (2.31)[a]	2.09 (3.24)[a]
Neonate	1.33	1.45 (9.02)[a]	1.31 (1.50)[a]	1.26 (5.26)[a]

[a] Relative difference to measurements via microscopy (%).

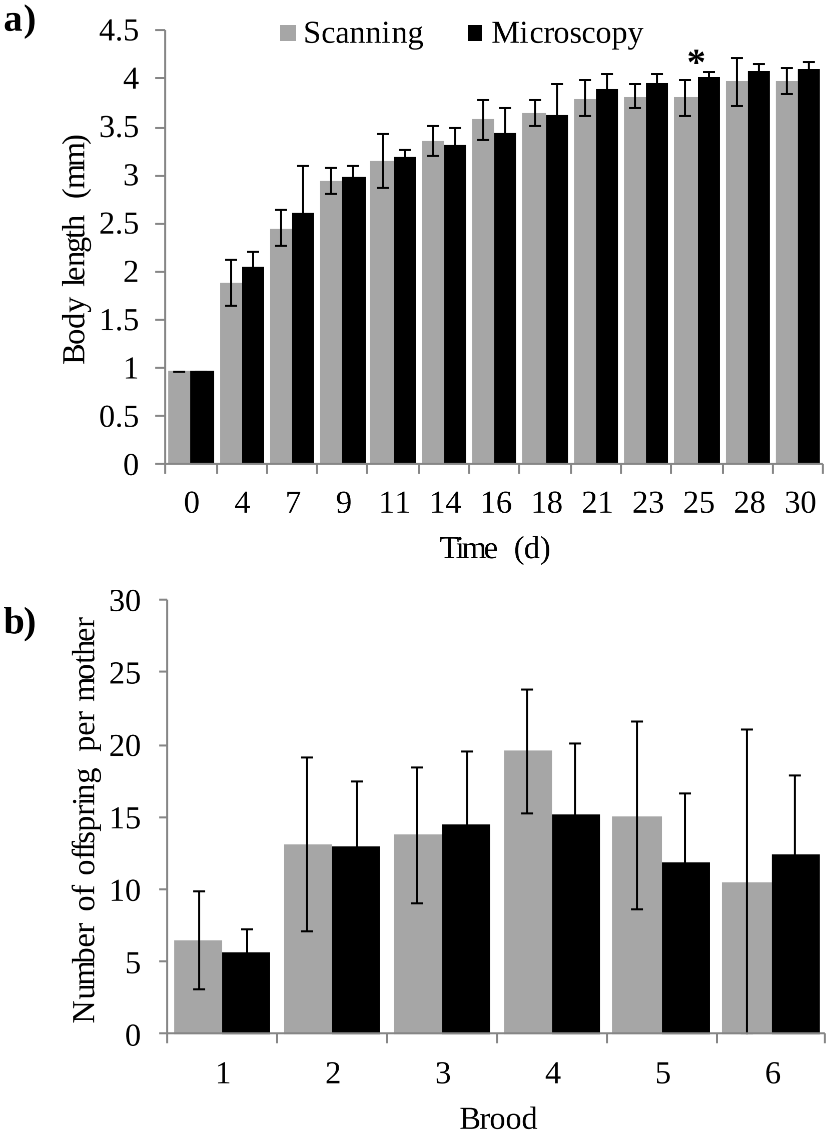

Figure 2: Comparison of growth (a) and reproduction (b) when using either microscopy or picture analysis for data acquisition. * Significant difference at indicated day (Two-way ANOVA, Holm-Sidak method, $p < 0.05$).

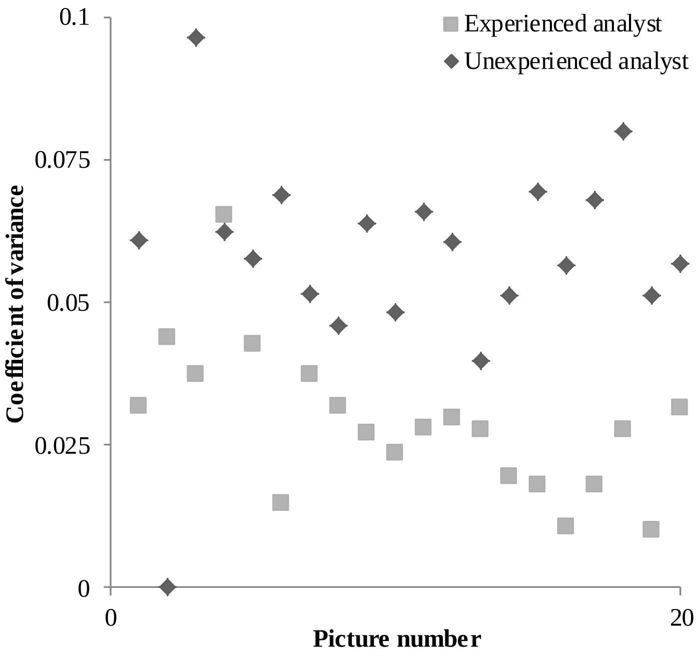

Figure 3: Coefficient of variance for 20 consecutively analysed pictures taken with the scanning method in dependence of the level of experience in picture analysis.

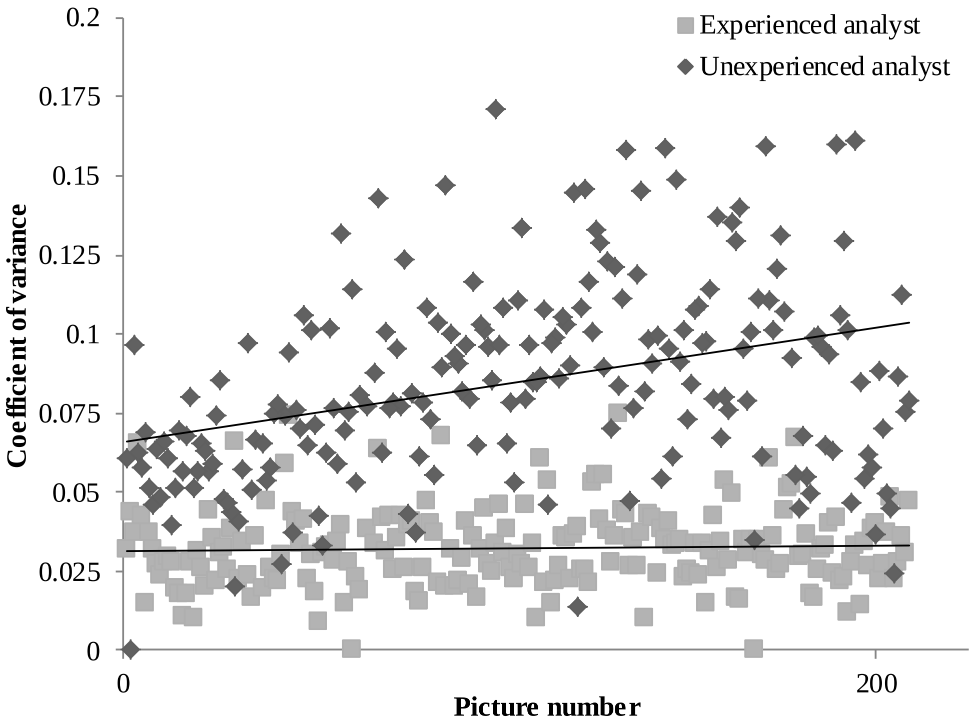

Figure 4: Coefficient of variance for > 200 consecutively analysed (within 10h) pictures taken with the scanning method in dependence of the level of experience in picture analysis.

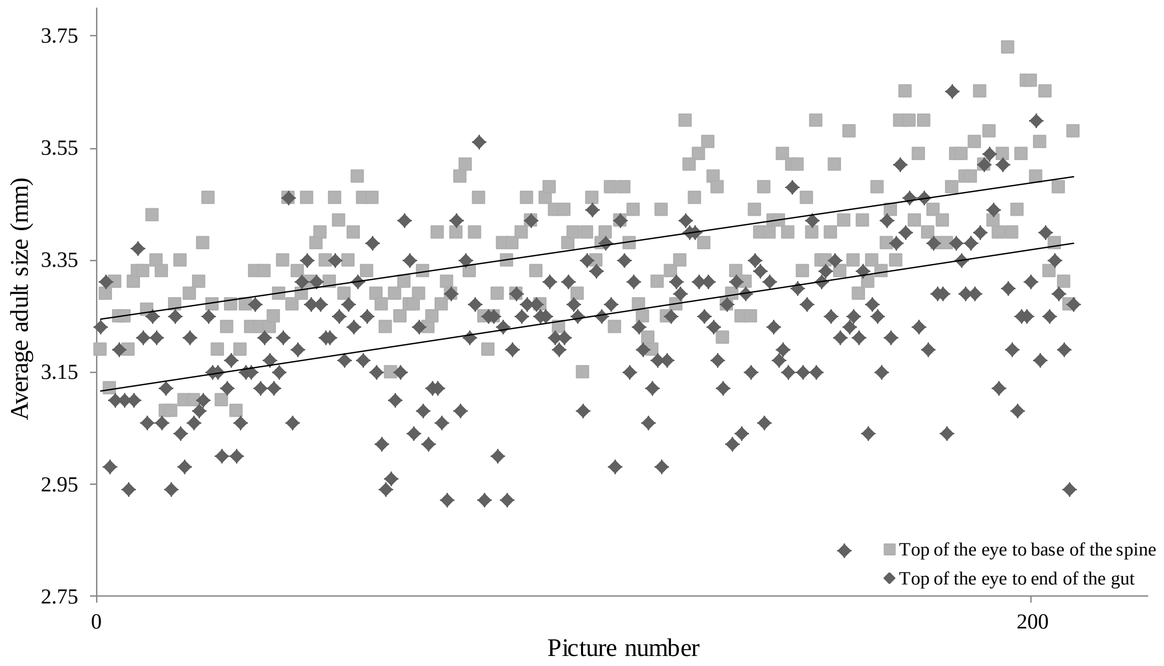

Figure 5: Measured adult size (mm) of *Daphnia magna* using two variations of the definition of body length.
