# Supplementary material for: Family-portraits for daphnids: scanning living individuals and populations to measure body length
Source: Ecotoxicology. 2015 Jun 6;24(6):1385–94. doi: 10.1007/s10646-015-1490-0 (PMC4515252; doi:10.1007/s10646-015-1490-0)
Supplement: Supplementary file 4 — Online Resource 4 (PDF 1108 kb) [file 10646_2015_1490_MOESM4_ESM.pdf]

This document will briefly explain how to add a new scanner to the programme and how to manually analyse a picture.

Note: The programme consists of two files “Measuring Body Length.exe” and “scanner.txt”. *“Measuring Body Length.exe”* is the actual interface to work with and “scanner.txt” is a text file where information on the scanner used is stored. Both files must be kept in the same sub-directory for the programme to work.

Adding a new standard measurement

1. Scan a millimetre scale faced down in a Petri dish using the preferred scanner settings and save the picture as a jpg-file.
2. Open the exe-file “Measuring Body Length.exe” located in the folder “Measurement”. Three windows called “Length Measurements”, “Image” and “Data” open.
3. In the window “Length Measurements” go to “File - Load Picture”. The picture will open in the window “Image” and the window name will display the subdirectory and the file name.
4. In the window “Length Measurements” go to “Image - Full Size”. The picture will be scaled to the original size.
5. In the window “Length Measurements” go to “Measurement - Standard”. Choose the preferred “cursor colour“ and “cursor size” and measure the standard by clicking with a single click on the beginning and end of 1 mm. The measurement will be shown in the data-sheet “Measurements” in the window “Data”. Repeat the measurement until a message indicating the number of pixels/mm opens.

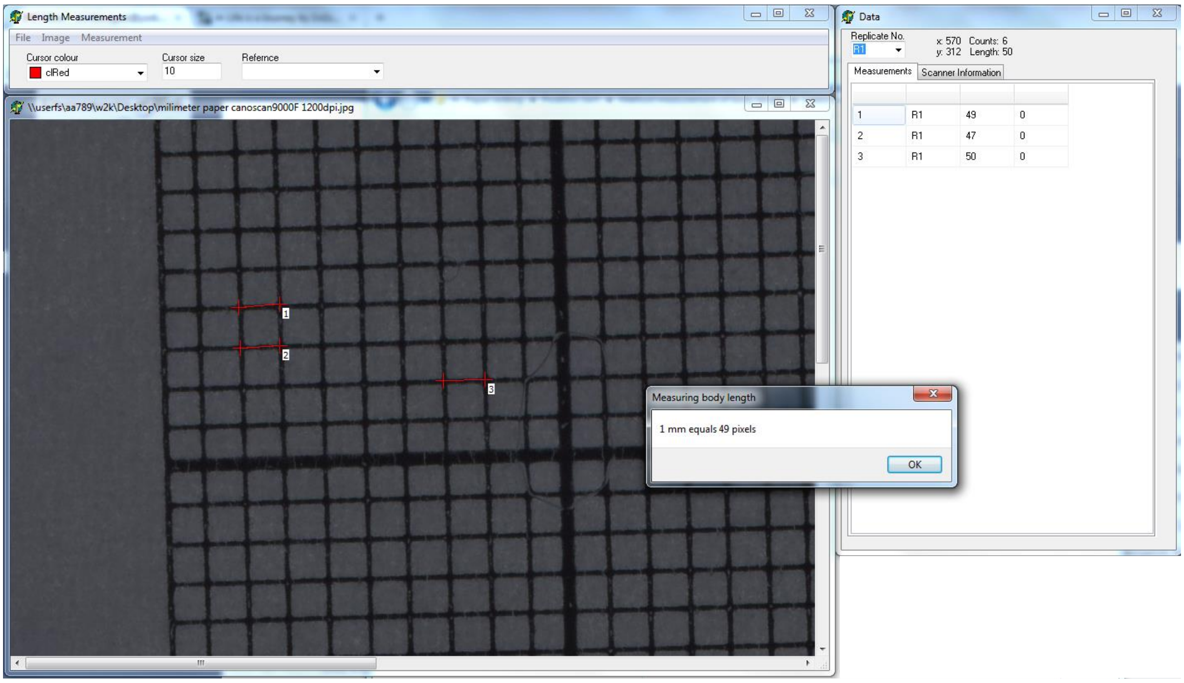

6. Close the programme and open the text-file "Scanner"
7. Add a new line with the following information **name;number** of pixels/mm (here: Demo;49).
8. Save the changes and close the text-file. Open the programme again. The new standard (here: Demo) will appear in the data-sheet <u>"Scanner Information"</u> and can be chosen as <u>"Reference"</u> in the <u>"Length Measurement"</u> window.

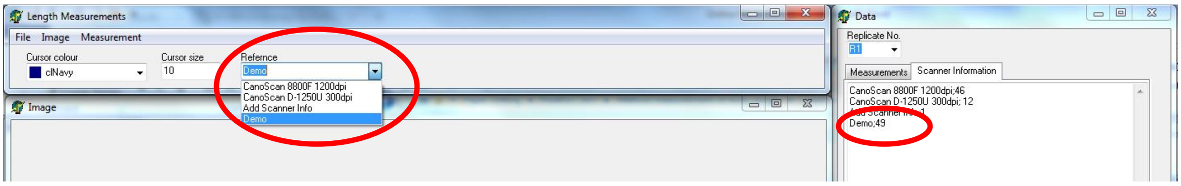

Analysing a picture

1) Open the picture to analyse: Go to "File - Load Picture" in the window "Length Measurement".
2) Scale the picture to its original size: Go to "Image - Full Size" in the window "Length Measurement".
3) Choose the preferred "cursor colour", "cursor size" and "Reference" in the "Length Measurement" window.
4) Chose the type of measurement to be conducted at "Measurement"

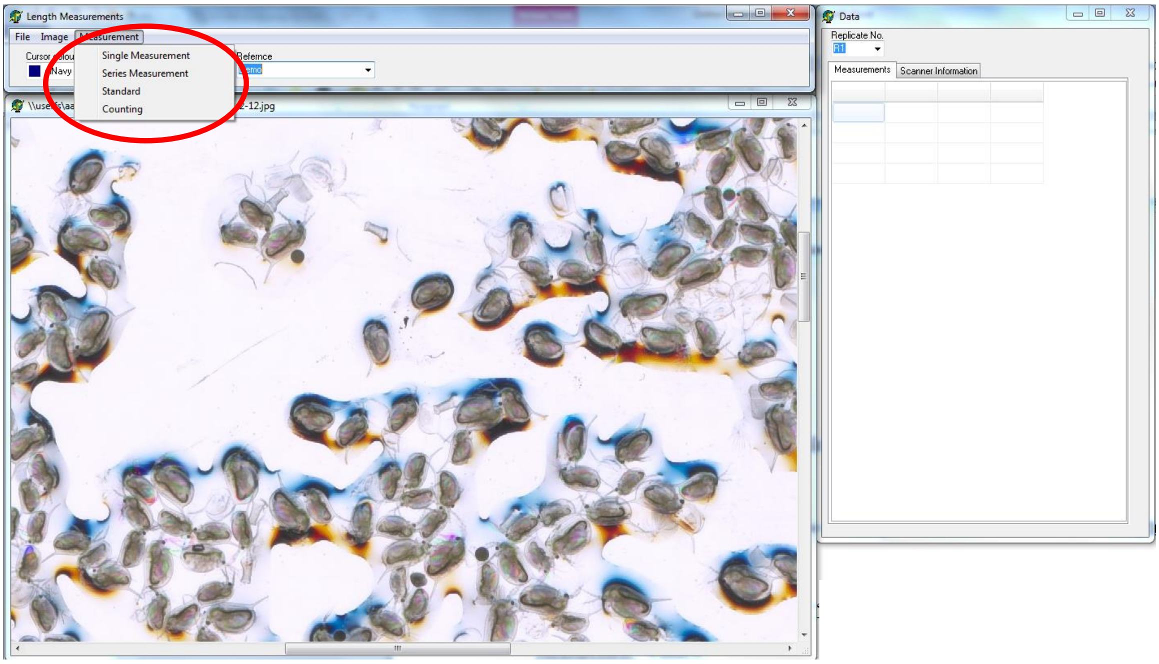

   i) Chose "Single Measurement" if the length of a single object is to be measured in one to numerous sub-sections (Example 1: measurement of the gut length).
   ii) Chose "Series Measurement" if the length of multiple objects is to be measured in single lines (Example 2: measuring the length of neonates).
   iii) Chose "Counting" if the number of objects is to be determined (Example 3: counting aborts).

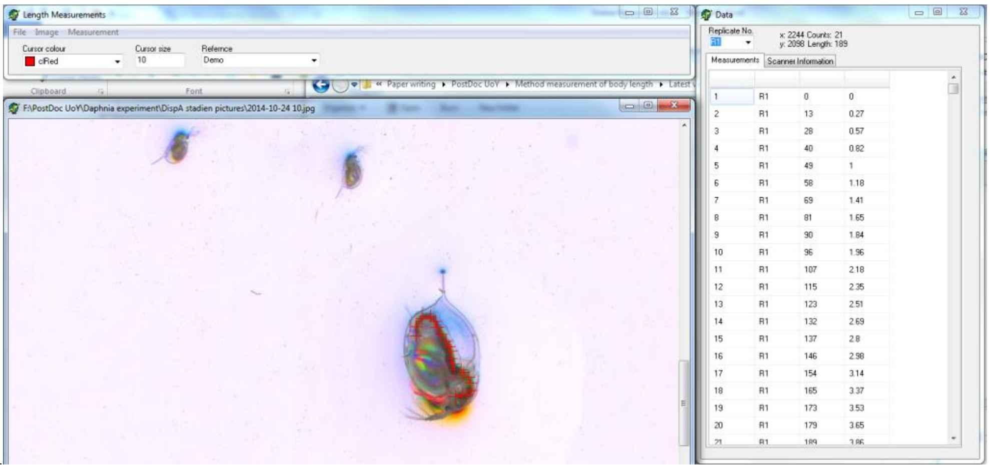

Example 1

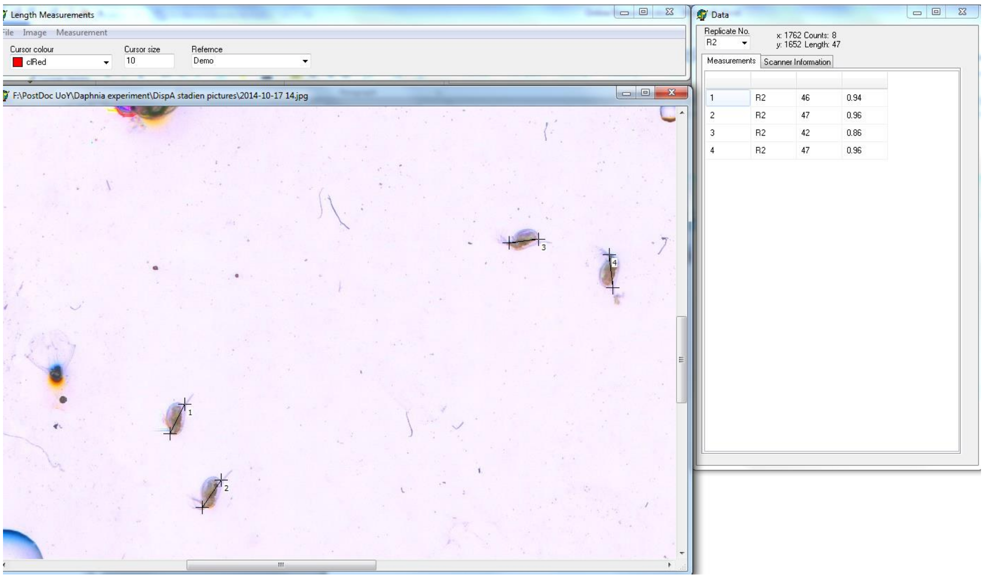

Example 2

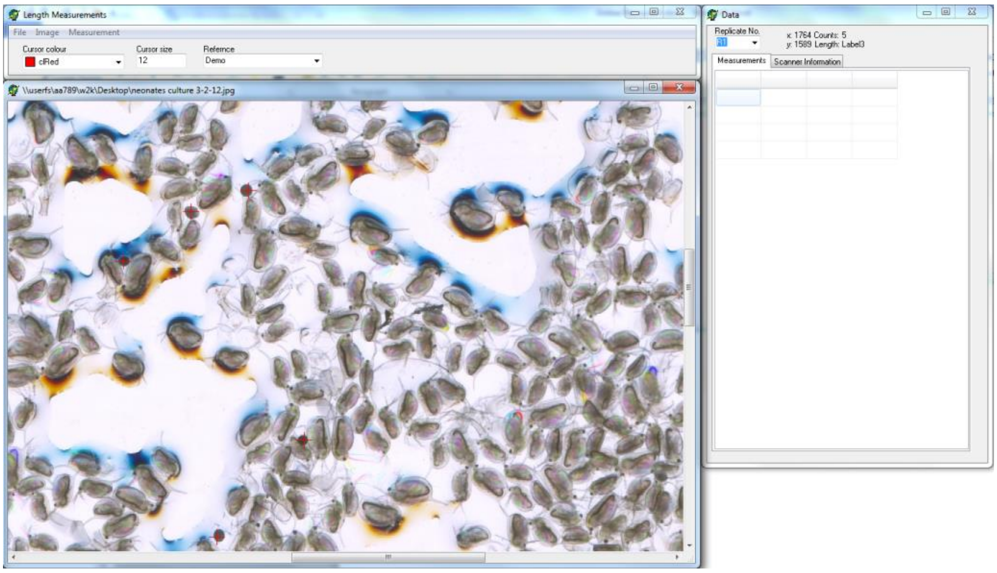

Example 3

5) Conduct all measurements. Measurements are shown in the window “Data” as follows:

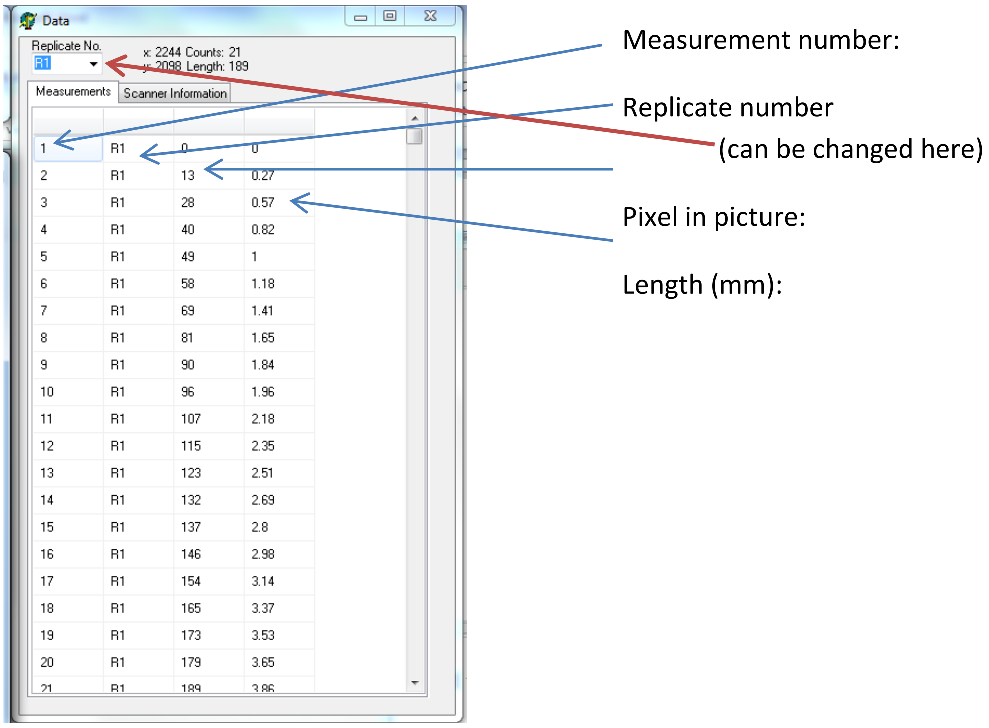

6) Saving generated data: Both, the picture including the illustration of the measurement and the data can be saved.

   i) To save the picture, go to “File – Save Picture” choose the directory and file name.

   ii) To save the generated data go to “File – Save Measurement” choose the directory and file name. **Note:** It is necessary to add **“.csv”** to the file name to save the data as excel readable file.

Note: The programme does allow the change of the measurement type (Series Measurement, Single Measurement, and Counting) but does start a new measurement with every change of measurement type. Generated data will be overwritten if not saved before the change of measurement type.

Several different types of length measurements can however be combined by changing the “Replicate No” in the window “Data”. This setting can also be changed to open a series of pictures after each other to store all data in one file. An example is given in the Example 4.

Example 4

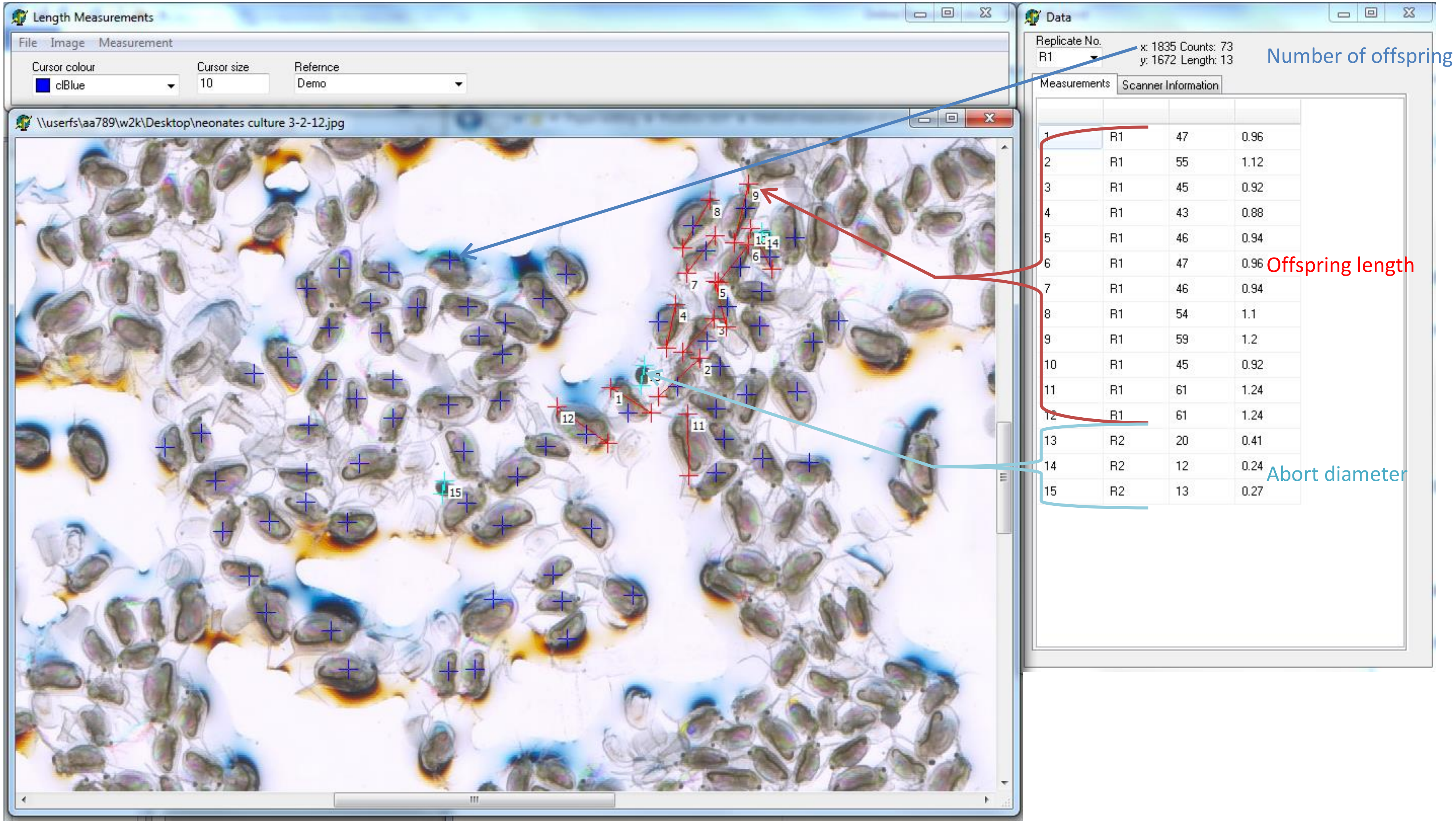
Length Measurements
File Image Measurement
Cursor colour
clBlue
Cursor size
10
Refernce
Demo
\\userfs\aa789\w2k\Desktop\neonates culture 3-2-12.jpg
Data
Replicate No.
R1
x: 1835 Counts: 73
y: 1672 Length: 13
Number of offspring
Measurements
Scanner Information
1 R1 47 0.96
2 R1 55 1.12
3 R1 45 0.92
4 R1 43 0.88
5 R1 46 0.94
6 R1 47 0.96
7 R1 46 0.94
8 R1 54 1.1
9 R1 59 1.2
10 R1 45 0.92
11 R1 61 1.24
12 R1 61 1.24
13 R2 20 0.41
14 R2 12 0.24
15 R2 13 0.27
Offspring length
Abort diameter
